# A Baseline Algorithm for Molecular Diagnosis of Genetic Eye Diseases: Ophthalmologist’s Perspective

**DOI:** 10.4274/tjo.59375

**Published:** 2016-12-01

**Authors:** Hande Taylan Şekeroğlu, Gülen Eda Utine, Mehmet Alikaşifoğlu

**Affiliations:** 1 Hacettepe University Faculty of Medicine, Department of Ophthalmology, Ankara, Turkey; 2 Hacettepe University Faculty of Medicine, Department of Pediatrics, Division of Pediatric Genetics, Ankara, Turkey; 3 Hacettepe University Faculty of Medicine, Department of Medical Genetics, Ankara, Turkey

**Keywords:** Genetic eye diseases, molecular diagnosis, gene therapy

## To the Editor:

Genetic eye diseases constitute a large and heterogeneous group. Individual diseases may cause multiple structural/functional anomalies and developmental features. Family history may be suggestive; however, it may also be challenging, particularly in late-onset conditions or in cases of variable expression.

In the current era of genetic advances, diagnosis of a genetic eye disease is facilitated by well-established collaboration between ophthalmologists and geneticists, as increasingly more patients will be asking for genetic counseling and prenatal diagnosis in addition to ophthalmologic management. Molecular investigation of a genetic eye disease requires customized analysis and advanced technology in addition to the requisite detailed family history and accurate ophthalmological diagnosis. A common indication for genetic testing is the validation of a preliminary diagnosis made in clinical practice. The need to determine the prognostic implications of the genotype, assessment of the recurrence risk and in particular, the possibility of specific gene therapy in the near future encourages clinicians to pursue genetic research.

**Figure 1:**
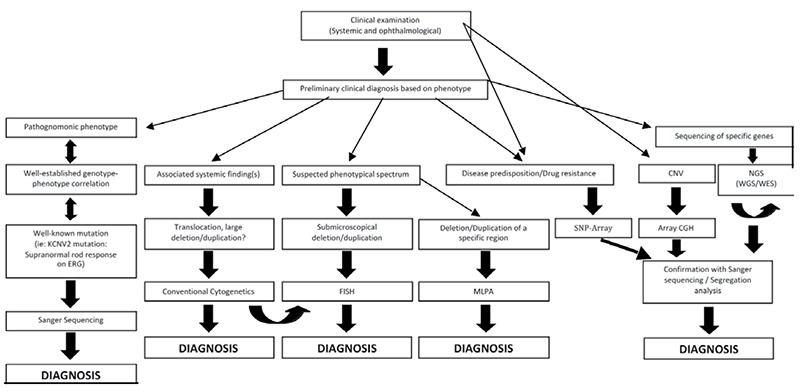
A baseline algorithm for the diagnosis of genetic eye diseases. CGH: Comparative genomic hybridization, CNV: Copy number variations, ERG: Electroretinogram, FISH: Fluorescence in situ hybridization, MLPA: Multiplex ligation-dependent probe amplification, NGS: Next generation sequencing, SNP: Single nucleotide polymorphism, WGS: Whole genome sequencing, WES: Whole exome sequencing

We present here a baseline algorithm covering common genetic mechanisms in order to outline a basic molecular approach for ophthalmologists. The first step of the flow chart, a prudent clinical examination with complete description of the phenotype, is indispensible for making a precise and accurate preliminary diagnosis (Figure 1). If the phenotype is pathognomonic, Sanger sequencing is preferred for confirmation.^1^ A previously established genotype-phenotype correlation may add to the value, either by providing accurate prognostic information or by indicating which particular mutation to look for. One such example may be electroretinographic supranormal rod response, indicating KCNV2 mutation type cone dystrophy, which can be precisely detected by Sanger sequencing or qPCR.^2^

Conventional karyotyping reveals microscopically visible abnormalities in chromosome number and structure, as well as translocations and large indels, and is appropriate as the first-tier test in multisystemic congenital abnormalities. Although conventional cytogenetic analysis may be considered as a screening test in such patients, microscopic diagnosis sometimes requires preliminary clinical diagnosis, designed in order to unveil specific deletions or duplications. A classic example is the small 11p interstitial deletion in Wilms tumor and aniridia, which could only be shown via fluorescence in situ hybridization or multiplex ligation-dependent probe amplification.

Array comparative genomic hybridization methods are preferred for genetic eye diseases involving copy number variations. One such example is congenital cataract, which has a very complicated phenotype-genotype correlation and shows clinical heterogeneity. Responsible mutations in crystallins, transcription factors and membrane proteins have been reported.^3^ Furthermore, single nucleotide polymorphism array may enable the detection of disease predisposition or drug resistance (e.g. age-related macular degeneration).

Next generation sequencing is the most current technology allowing parallel sequencing of many genes and may cover either a spectrum of known genes or all exons of all genes, allowing the discovery of new causative genes. The latter is called whole exome sequencing, and is a popular and practical investigation tool for developmental diseases.^1^ Genetic testing, theoretically, can also reveal the underlying ocular problem in cases with subnormal vision but otherwise normal ophthalmological examination (i.e. inherited retinal dystrophies), or it can define the high-risk group for an ocular disease and factors that prevent/delay any poor prognosis (i.e. early-onset glaucoma).^4^

The ultimate aim is to treat the condition. This is crucial in genetic disorders, in which modern treatment suggestions involve replacement of the missing molecular element. Many ongoing trials regarding gene therapies appear to have promising results for future treatment options.^5^ Ophthalmologists would benefit from a practical flow chart based on a priori assumption of genetic basis for each genetic eye disease. This would not only save time and money but may also lead to practical advances in diagnosis and management.
